# Improved variance estimation of classification performance via reduction of bias caused by small sample size

**DOI:** 10.1186/1471-2105-7-127

**Published:** 2006-03-13

**Authors:** Ulrika Wickenberg-Bolin, Hanna Göransson, Mårten Fryknäs, Mats G Gustafsson, Anders Isaksson

**Affiliations:** 1 Department of Genetics and Pathology, Uppsala University, Rudbeck Laboratory, SE-751 85 Uppsala, Sweden; 2 Department of Engineering Sciences, Uppsala University, Box 528, SE-751 20 Uppsala, Sweden

## Abstract

**Background:**

Supervised learning for classification of cancer employs a set of design examples to learn how to discriminate between tumors. In practice it is crucial to confirm that the classifier is robust with good generalization performance to new examples, or at least that it performs better than random guessing. A suggested alternative is to obtain a confidence interval of the error rate using repeated design and test sets selected from available examples. However, it is known that even in the ideal situation of repeated designs and tests with completely novel samples in each cycle, a small test set size leads to a large bias in the estimate of the true variance between design sets. Therefore different methods for small sample performance estimation such as a recently proposed procedure called Repeated Random Sampling (RSS) is also expected to result in heavily biased estimates, which in turn translates into biased confidence intervals. Here we explore such biases and develop a refined algorithm called Repeated Independent Design and Test (RIDT).

**Results:**

Our simulations reveal that repeated designs and tests based on resampling in a fixed bag of samples yield a biased variance estimate. We also demonstrate that it is possible to obtain an improved variance estimate by means of a procedure that explicitly models how this bias depends on the number of samples used for testing. For the special case of repeated designs and tests using new samples for each design and test, we present an exact analytical expression for how the expected value of the bias decreases with the size of the test set.

**Conclusion:**

We show that via modeling and subsequent reduction of the small sample bias, it is possible to obtain an improved estimate of the variance of classifier performance between design sets. However, the uncertainty of the variance estimate is large in the simulations performed indicating that the method in its present form cannot be directly applied to small data sets.

## Background

It is crucial to show that a classifier designed using supervised learning performs sufficiently well for the application of interest. A minimum requirement is that it is performs better than random guessing. Recently gene expression profiling using microarray technology has been widely used for classification of tumors based on supervised learning [1-3]. Various cross-validation and resampling methods aimed at providing reliable and robust estimates of classifier performance have been proposed [4,5]. A natural measure of the robustness of an algorithm is the variance of the distribution of error rates when the classifiers are designed using the number of training examples available. Recently attempts have been made to obtain confidence intervals based on small sample sizes [6,7]. These approaches correspond to an idealized case where the bounds on the unknown performance of a classifier designed using *N*_*d *_samples are obtained by repeated designs and tests using new examples. If this procedure would yield a large set of high quality performance estimates, their distribution could be used to estimate a 95% confidence interval (CI) of the true error rates. Notably, in this approach no point estimate of the performance for the particular classifier of interest is calculated. The quantity of interest is the 95% CI for the whole distribution of possible true performances. Since this CI covers the true performance of interest with probability 95%, without any additional information available, e.g. from a conventional holdout test, it represents the current state of uncertainty about the true performance.

Since estimation of CI using this method would require access to large amounts of data that are not available in practice, a suggested alternative approach is to estimate the CI using resampling techniques like in the recent work by Michiels *et al*. [6]. In their work, a performance estimation method called repeated random sampling (RRS), that was originally described by Mukherjee *et al*. [7], is applied to seven large gene expression data sets. For almost all the data sets, Michiels *et al*. demonstrate that the sizes of the CIs obtained from the RRS procedure increase with increasing sizes of the design sets. This is counterintuitive as the variance *σ*_*d*_^*2 *^of the true performances should decrease with increasing size of the design sets. With more data used for design, the placement of the decision boundary of the classifier will be more stable and as a consequence the resulting *σ*_*d*_^*2 *^will be lower [4]. Hence, the variance and confidence interval obtained from RSS often have a bias.

In this paper we identify small test-set size as one factor that can lead to the bias in the variance estimate observed using RRS. We also introduce a first order model of the variance estimate as a function of the number of test examples for a refined, less biased, estimation method called Repeated Independent Design and Test (RIDT). Furthermore, we demonstrate that by modeling the undesirable small sample bias in RIDT, it is possible to greatly reduce the bias in the estimates of *σ*_*d*_^*2 *^and therefore in the resulting CIs. For the special case of repeated designs and tests using completely novel samples, we present an exact analytical expression for how the bias in the estimates of *σ*_*d*_^*2 *^decreases with increasing size of the test sets.

## Results

### The estimated variance in repeated cross-validation depends on the number of test data

**Figure 1:**
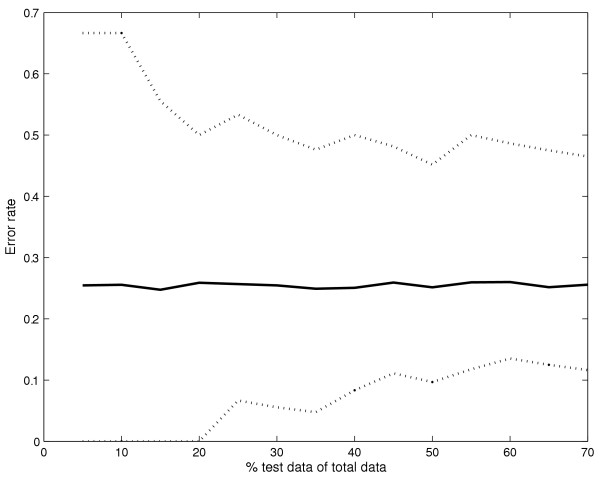
**Repeated random sampling with different test set sizes**. Results from repeated random sampling where the size of the design sets was set to 30% of the total sample size and the size of the test set was varied from 5% to 70%. For each test set size the data was divided randomly into design and test sets 1,000 times, with the class proportions kept constant. The endpoints (dotted) of a two-sided 95% CIs, based on a histogram of 1,000 estimates, is displayed for the different values of the test set size, *N*_*t*_. Apparently the widths of the empirical CIs decrease as N_t _increases. Also displayed are the estimated averages as a function of the test set size (solid). Since each CI is based on the histograms instead of estimates of the average and variance, note that the CIs are asymmetric with respect to the estimated average.

Fukunaga and Hayes [8] pointed out that small test set size *N*_*t *_affects the variance of the performance estimates obtained in repeated hold out experiments. This variance may be regarded as an estimate of *σ*_*d*_^*2*^and is denoted ^*RH*^*σ*_*dt*_^2 ^to indicate that it depends on *N*_*t*_. We argued that a similar effect may also affect similar repeated cross-validation methods including RRS. Since the total number of examples is fixed, the number of test examples is automatically decreased when the size of design data is increased in RRS. To be able to study the effect of test data size on its own, we modified the RSS procedure and kept the size of the design data constant while varying the size of the test data. We used a colon cancer microarray data set containing 22 normal and 40 colon cancer cases [9] and classified the samples using a modified Fisher's linear discriminant classification algorithm (see Methods). The size of the design was constant at 30% of the total data set size, while the size of the test set, *N*_*t *_was varied in steps from 70%, (identical to RRS using all data), down to 5%. For each value of *N*_*t*_, the original data set was divided randomly into design and test sets 1,000 times, while maintaining class proportions. An almost trivial fact discussed formally by Fukunaga and Hayes [8] is that the mean *m*_*d *_and the variance *σ*_*d*_^*2 *^of the distribution of true error rates is independent of the number of test examples *N*_*t*_. Consequently, the CIs for the distribution of true error rates are constant for every choice of the number of design examples *N*_*d*_. The results in Figure 1 clearly show that the sizes of the CIs obtained using repeated cross-validation are not constant but decreasing with increasing values of *N*_*t*_. This is similar to what can be observed using RRS in Michiels *et al*. [6] where the number of design examples (and consequently also the number of test examples) is varied. As shown in Figure 1 the estimated CI stabilizes as the size of the test set becomes large. The bias in the CI will be eliminated as the size of the test set becomes very large. However, for the usually limited set of examples available in most real world problems, the bias is too large to be neglected.

### Repeated independent design and test

Limited testing of each classifier is not expected to be the sole cause of undesirable bias in the RSS estimate. Bias may also be attributed to three different statistical dependencies between data sets caused by the repeated design and testing performed using the bag of limited examples available: 1) Each pair of design and test sets are dependent. Once the design set has been selected, the remaining examples become the test set deterministically. 2) The design sets are inter-dependent. Given information about the samples in a first design set, a lot of information is gained about the possible samples that may occur in the next design set obtained by means of resampling. 3) The test sets are also inter-dependent. Given information about the samples in a first test set, information has been gained about the possible samples that may occur in the next test set. In this work we introduce a novel procedure denoted Repeated Independent Design and Test (RIDT), which eliminates the first type of dependence by splitting the original data set of size *N *into a design bag with *N*_*D *_samples, and a test bag, with *N*_*T *_= *N-N*_*D *_test samples. Thus, for each design a fixed number of examples *N*_*d *_with equal number of samples from each class are drawn with replacement form the bag of *N*_*D *_samples. This makes the resampling of design examples completely independent of the selection of test set examples. Notably, the design sets remain inter-dependent due to the small design bag and similarly the test sets remain inter-dependent due to the finite size of the test bag. By repeatedly selecting design sets of size *N*_*d *_from the design bag and testing with data from the test bag, a number of error rate estimates are obtained that subsequently are used to obtain an almost unbiased estimate of the true variance *σ*_*d*_^*2*^. This variance estimate can in turn be used to construct the desired CI of the distribution of true performances.

### A variance model for the RIDT procedure

Analogous to the variance ^*RH*^*σ*_*dt*_^*2 *^associated with idealized repeated holdout experiments discussed above, the variance of the error rate estimates obtained with RIDT is dependent on the finite value of N_t_, as well as on N_T _and is denoted *σ*_*dt*_^*2*^. To study and reduce estimation biases caused by small sample size, we propose that for a given data set *D*, the RIDT estimate of *σ*_*dt*_^*2 *^may be approximated as

σ^dt2(1NT,1Nt,D)≈α0(D)+α1(D)NT+α2(D)Nt.     Eq. (1)
 MathType@MTEF@5@5@+=feaafiart1ev1aaatCvAUfKttLearuWrP9MDH5MBPbIqV92AaeXatLxBI9gBaebbnrfifHhDYfgasaacH8akY=wiFfYdH8Gipec8Eeeu0xXdbba9frFj0=OqFfea0dXdd9vqai=hGuQ8kuc9pgc9s8qqaq=dirpe0xb9q8qiLsFr0=vr0=vr0dc8meaabaqaciaacaGaaeqabaqabeGadaaakeaaiiGacuWFdpWCgaqcamaaDaaaleaacqWGKbazcqWG0baDaeaacqaIYaGmaaGcdaqadaqaamaalaaabaGaeGymaedabaGaemOta40aaSbaaSqaaiabdsfaubqabaaaaOGaeiilaWYaaSaaaeaacqaIXaqmaeaacqWGobGtdaWgaaWcbaGaemiDaqhabeaaaaGccqGGSaalcqWGebaraiaawIcacaGLPaaacqGHijYUcqWFXoqydaWgaaWcbaGaeGimaadabeaakiabcIcaOiabdseaejabcMcaPiabgUcaRmaalaaabaGae8xSde2aaSbaaSqaaiabigdaXaqabaGccqGGOaakcqWGebarcqGGPaqkaeaacqWGobGtdaWgaaWcbaGaemivaqfabeaaaaGccqGHRaWkdaWcaaqaaiab=f7aHnaaBaaaleaacqaIYaGmaeqaaOGaeiikaGIaemiraqKaeiykaKcabaGaemOta40aaSbaaSqaaiabdsha0bqabaaaaOGaeiOla4IaaCzcaiaaxMaacqqGfbqrcqqGXbqCcqqGUaGlcqqGGaaicqqGOaakcqqGXaqmcqqGPaqkaaa@607E@

This equation involves first order linear approximations of the biases introduced by the finite values of *N*_*T *_and *N*_*t *_(see Methods). For very large values of *N*_*T *_and *N*_*t*_, the estimate reduces to an unbiased estimate of *σ*_*d*_^*2*^*(N*_*d*_*)*. Hence the first coefficient *α*_*0*_*(D) *should be an unbiased estimate of *σ*_*d*_^*2*^*(N*_*d*_*)*, i.e. <*α*_*0*_*(D) >*_*D *_= *σ*_*d*_^*2 *^*(N*_*d*_*) *where *<>*_*D *_denotes the expectation operator. Notably, the model treats the size of the test bag *N*_*T *_in a similar way as the size of the test set *N*_*t*_, but ignores effects due to size of the design bag *N*_*D*_.

By evaluating classifications using *N*_*b *_design sets, varying the test bag sizes *N*_*T *_and sizes of tests sets *N*_*t *_for each value of *N*_*b*_, it is possible to estimate the data set dependent coefficients *α*_*0*_*(D)*, *α*_*1*_*(D) *and *α*_*2*_*(D) *in Eq. (1) by multivariate least squares fitting. In this process one constraint is used that ensures the natural inequality *α*_*0*_*(D) *≥ 0. With access to the fitted coefficient *α*_0_*(D) *one has obtained an unbiased estimate of the desired quantity *σ*_*d*_^*2*^*(N*_*d*_*).*

We performed simulations using samples generated from two 2-dimensional normal distributions with mean values and covariance matrices estimated from real micorarray gene expression data [9]. The aim was to validate our model, and to demonstrate its potential for elimination of the bias caused by small sample size (see Methods). Since the two features (artificial gene activities) in both distributions are correlated, the simulation takes the dependence that may exist between features (genes) in real data sets into account. One should also note that we have deliberately chosen to use a classifier that does not contain a feature selection step to avoid additional complexity. Thus, the problems and solution discussed in this paper are equally relevant also for classifier using feature selection. We have also chosen the strategy to evaluate the performance for each class separately, since it does not require knowledge about the probabilities of observing examples from class 1 or class 2.

**Figure 2:**
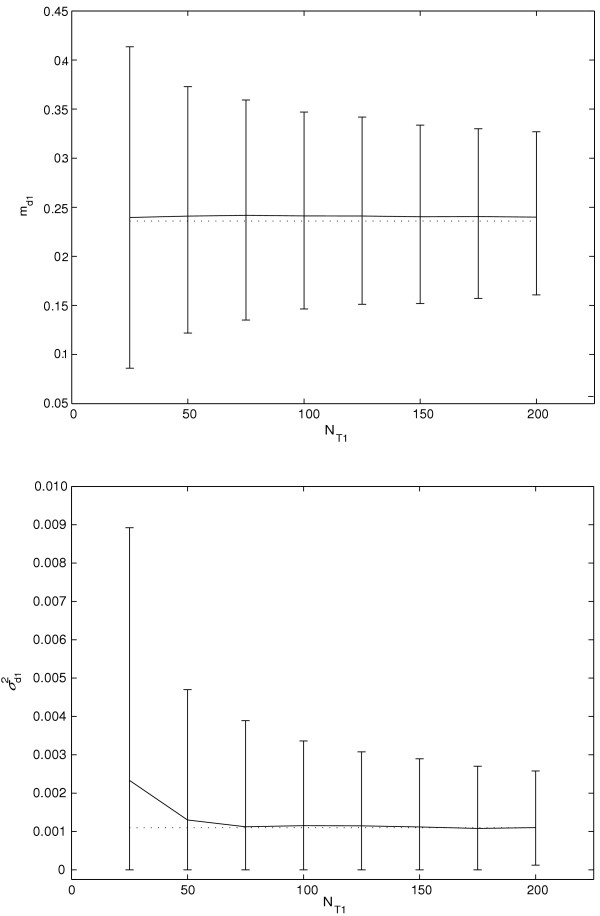
**Unbiased estimates of *m*_*d1 *_(top) and *σ*_*d1*_^*2 *^(bottom)**. The true values of *m*_*d1 *_and *σ*_*d1*_^*2 *^(dotted) are compared to the average of 50 conservative point estimates of *m*_*d1 *_and *σ*_*d1*_^*2 *^(solid) for the 2-dimensional normal distributions used where *N*_*d *_= 100. Also displayed are two-sided 95% CIs, based on histograms of the 5000 estimates for different values of the test bag size *N*_*T1*_. The true *m*_*d1 *_and *σ*_*d1*_^*2 *^were obtained by testing 10,000 independently designed classifiers using 100,000 new, independently generated, test samples from the two normal distributions used. The estimates of *m*_*d1 *_are unbiased and the estimates of *σ*_*d1*_^*2 *^are unbiased for *N*_*T1 *_≥ 100.

To emphasize that we focused the analysis on class 1, an extra subindex was introduced when denoting the quantities of interest. Thus, the true mean value and variance associated with class 1 are denoted *m*_*d1 *_and *σ*_*d1*_^*2*^, respectively, and corresponding quantities associated with testing using finite data sets are denoted *m*_*dt1 *_and *σ*_*dt1*_^*2*^. In the RIDT procedure the design bags had equal number of samples from both classes and the number of samples drawn with replacement was the same as the size of the design bag, i.e. *N*_*d *_= *N*_*D*_. We also made the assumption that *m*_*dt1 *_is an unbiased estimate of *m*_*d1 *_which was verified (Figure 2).

The mean values of the estimated *m*_*d1 *_and *σ*_*d1*_^*2*^, and the corresponding two-sided 95% CIs for eight different values of *N*_*T1 *_(*N*_*T1 *_= 25, 50, 75, ..., 200), are presented in Figure 2 for *N*_*d *_= *N*_*D *_= 100 . The true values *m*_*d1 *_and *σ*_*d1*_^*2*^, obtained by testing 10,000 independently designed classifiers using 500,000 test samples each, are also indicated. Apparently, unbiased estimates of *m*_*d1 *_and *σ*_*d1*_^*2 *^are obtained.

We observed that the reduction of the small sample bias yields accurate estimates of *σ*_*d1*_^*2 *^on average. One should note that in general the estimates contain contributions of higher order terms. However, as the results indicate, the biases caused by these higher order terms may be quite small for commonly used sizes of data sets. For the data set sizes commonly used it appears that it is possible to obtain practically unbiased estimates of *m*_*d1 *_and *σ*_*d1*_^*2 *^with the RIDT method.

### Variance for independent data

In the special case of truly independent data, i.e. when each pair of design and test sets are drawn from the underlying true distribution of samples instead of from a finite bag of examples, we derived an exact analytical equation for *σ*_*dt*_^*2 *^(see Additional File 1):

σdt2=σd2+md(1−md)−σd2Nt.     Eq. (2)
 MathType@MTEF@5@5@+=feaafiart1ev1aaatCvAUfKttLearuWrP9MDH5MBPbIqV92AaeXatLxBI9gBaebbnrfifHhDYfgasaacH8akY=wiFfYdH8Gipec8Eeeu0xXdbba9frFj0=OqFfea0dXdd9vqai=hGuQ8kuc9pgc9s8qqaq=dirpe0xb9q8qiLsFr0=vr0=vr0dc8meaabaqaciaacaGaaeqabaqabeGadaaakeaaiiGacqWFdpWCdaqhaaWcbaGaemizaqMaemiDaqhabaGaeGOmaidaaOGaeyypa0Jae83Wdm3aa0baaSqaaiabdsgaKbqaaGGaaiab+jdaYaaakiabgUcaRmaalaaabaGaemyBa02aaSbaaSqaaiabdsgaKbqabaGccqGGOaakcqaIXaqmcqGHsislcqWGTbqBdaWgaaWcbaGaemizaqgabeaakiabcMcaPiabgkHiTiab=n8aZnaaDaaaleaacqWGKbazaeaacqaIYaGmaaaakeaacqWGobGtdaWgaaWcbaGaemiDaqhabeaaaaGccqGGUaGlcaWLjaGaaCzcaiabbweafjabbghaXjabb6caUiabbccaGiabbIcaOiabbkdaYiabbMcaPaaa@52CC@

This equation shows how the observed variance depends on the number of test samples *N*_*t *_as well as on *m*_*d *_and *σ*_*d*_^*2*^. It can also be noted that for very large values of *N*_*T*_, Eq. (1) reduces to the same form as Eq. (2). Fukunaga and Hayes [8] have previously published an approximation of Eq (2). One advantage with the exact equation is that it can be used to show that the second term always is larger than zero and that *σ*_*dt*_^*2 *^is always larger than *σ*_*d*_^*2*^. Thus, if *σ*_*dt*_^*2 *^would be an approximation of the variance, the resulting CI would always be conservative.

**Figure 3:**
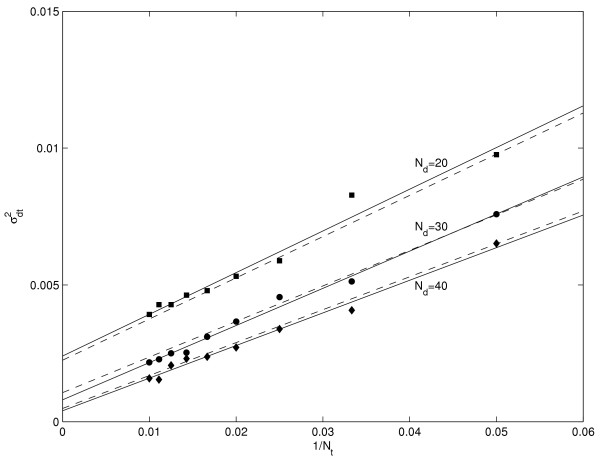
**Least squares fitting for different sizes of *N*_*d*_**. The variance *σ*_*dt*_^*2 *^plotted against *1/N*_*t*_, where *N*_*t *_is the number of test (holdout) samples used. The solid lines are the least squares fittings of the simulated average values for 1/*N*_*t*_. The dashed lines are produced by means of the analytical result in Eq. (2).

In order to empirically validate Eq. (2), simulations were performed using samples drawn from two 8-dimensional normal distributions (see Methods). We determined *σ*_*dt*_^*2 *^for different values of *N*_*t *_and *N*_*d *_(see Methods). Each value of *σ*_*dt*_^*2 *^was obtained using a histogram of 1,000 independent point estimates (Figure 3). For comparison, 1,000 separate and independent high accuracy point estimates of *m*_*d *_and *σ*_*d*_^*2 *^were computed, each using 10,000 test samples for varied design set sizes *N*_*d*_. The true observed variances in Figure 3 were then obtained from Eq. (2). This demonstrates excellent agreement between the theory and simulations.

## Discussion

RRS has been proposed as a practical method for estimation of the distribution of error rates obtained when a specified number of data samples are used for design [6,7]. However, we have demonstrated that the variance estimate of the performance for classifiers designed and tested in a similar way results in a variance estimate that is highly dependent on the number of samples used for test (Figure 1). A qualitatively analogous effect should occur also in RRS, which is equivalent to using all remaining examples for test in our experiment. Consequently, highly conservative estimates of the variance are obtained with repeated testing methods when the number of examples used for test is small. In practice the variance estimates have a bias of unknown magnitude, due to the complex statistical dependence between design and test sets. Therefore, it is important to stress that the confidence interval in RRS cannot be used to draw any conclusions about whether it is likely that a classifier performs better than chance. An example of this inappropriate use of RRS can be found in [4] where the possibilities to predict cancer outcome based on microarray gene expression patterns were investigated in several data sets.

Perhaps even more importantly, a large bias in the variance estimate of interest is not a unique feature of the RSS procedure but is expected to be found in all other suggested resampling procedures for performance estimation. For example, estimating the variance of a q-fold CV performance estimate as suggested by McLachlan *et al*. [10] (page 216) seems attractive but we are not aware of any theoretical or numerical proofs that those and similar methods result in unbiased estimates of the variance *σ*_*d*_^*2*^of interest. On the contrary, the proof of Equation (2) in our manuscript clearly shows that even if it would be possible to draw infinitely many independent design and test sets from the true distribution of samples, the resulting variance estimate of interest is heavily biased when the test sets are small.

There are a number of features of the RIDT method that have implications for the use of the method. First, the RIDT performance estimates rely on a split of the data set into two separate parts, one used for repeated design, the other for repeated tests, which is not current practice in cross-validation and bootstrapping and might be interpreted as inefficient use of the few samples available. We view this as a price that has to be paid in order to provide unbiased estimation of the variance of interest which can not be obtained with other methods. Second, although normal distributions were used in the computer simulations performed to generate the results presented here, the elimination of finite sample effects using Eq. (1) does not assume normally distributed data, but can use data from any type of distribution. Third, even though Eq. (1) does not depend on *N*_*D*_, it is possible to reduce small sample effects and provide unbiased estimates for the specific problem considered here. The general applicability of this observation awaits further studies but Eq. (1) can easily be extended to include a fourth term that is explicitly dependent on *N*_*D*_, see Eq. (7). One possible explanation for the small influence of *N*_*D *_in the RIDT method used here is that the design sets are drawn with replacement from the design bag, a procedure that closely reflects what happens in reality.

Although not yet explored in detail, there are several explanations for the small bias in *σ*_*d1*_^*2 *^observed in Figure 2 when *N*_*T1 *_≤ 50: 1) We are ignoring higher order terms in the approximations. 2) We do not try to eliminate effects caused by a finite value of *N*_*D*_. 3) We do not take any small sample effects into account at all when estimating *m*_*d*_. 4) When using replacement, we employ on average only 63.2% unique samples in each design [11]. Notably, the number of design examples *N*_*d *_remains fixed and, as discussed above, there is no contribution to the bias due to *N*_*d *_being small.

We find that the variance of the inter-design set variance estimate *σ*_*d1*_^*2 *^is relatively large and increases with decreasing value of *N*_*T1*_. This means that the estimate of *σ*_*d*_^*2 *^for a particular data set is unbiased, but that it may be associated with a large uncertainty especially if the size of the data set is small. Therefore, it is difficult to directly use the unbiased estimates of *m*_*d *_and *σ*_*d*_^*2 *^to construct a CI. Thus it appears that even though we can compensate for biases caused by small sample size, the resampling approach has not provided a method that is practically useful in its present form. Therefore the only rigorous option for estimation of classifier performance that we know of is the classical hold out test combined with a Bayesian credibility interval [12], even though this interval is overly conservative and provides very wide intervals.

## Conclusion

One major suggestion from the results of this paper is that previously introduced resampling and cross-validation methods for performance estimation using small sample sets are expected to result in large biases in their estimates of the inter design set variances. Consequently such biased variance estimates lead to inappropriate confidence intervals for the performance of a chosen classifier. In addition this paper describes a method that is capable of eliminating this bias for a new resampling method (RIDT) also introduced here. Finally we would like to point out that although this paper provides important experimental and theoretical results, the large variability in the unbiased variance estimate obtained still leaves us one step away from a practically useful solution for small sample based estimation of confidence intervals using resampling. We therefore also hope that this work will inspire others to consider how to convert the unbiased but highly variable variance estimate of our and similar future procedures into a valid confidence interval.

## Methods

### Observed variance from repeated designs and tests using the colon data

We used a colon cancer microarray data set, containing expression levels for 2,000 genes for 22 normal and 40 colon cancer cases [9]. The size of the design sets N_d _was set to 30% of the total sample size and the size of the test set was varied in steps from 5% to 70%. With the class proportions kept constant, the data was divided randomly into design and test sets 1,000 times for each test set size. A modified version of Fisher's linear discriminant classification algorithm [13] that is made more robust against small sample sizes was employed (Matlab code is available from the authors upon request). The classification algorithm was used together with the greedy pairs algorithm [14] for selection of four genes as previously described [15]. Since the exact procedure for the classifier design including the gene selection is of secondary interest in this study, an unbiased selection of the number of genes was not performed.

### Derivation of the variance model for the RIDT procedure

Consider the case where the number of samples in the data set, *D*, is small. To avoid dependence between design and test, the samples are first divided into a design bag with *N*_*D *_samples and a test bag with *N*_*T *_samples. Without loss of generality for illustration of how the variance can be modelled and the bias eliminated, we assume that the classification of one class is of main interest and that the test bag only includes *N*_*T1 *_samples from one class, i.e. *N*_*T *_= *N*_*T1*_. We consider the case where the number of samples in the design, *N*_*d*_, is the same as the number in the design bag, *N*_*D*_. Now consider the design of *N*_*b *_classifiers using repeated random sampling for the design bags. Each classifier is designed with *N*_*d *_samples, drawn with replacement from a design bag, and is then tested with *N*_*t1 *_test samples drawn without replacement from a test bag containing *N*_*T1 *_samples. *N*_*b *_error rate estimates are obtained, denoted

e^1b=e^1b(ND,NT1,Nt1,D)     Eq. (3)
 MathType@MTEF@5@5@+=feaafiart1ev1aaatCvAUfKttLearuWrP9MDH5MBPbIqV92AaeXatLxBI9gBaebbnrfifHhDYfgasaacH8akY=wiFfYdH8Gipec8Eeeu0xXdbba9frFj0=OqFfea0dXdd9vqai=hGuQ8kuc9pgc9s8qqaq=dirpe0xb9q8qiLsFr0=vr0=vr0dc8meaabaqaciaacaGaaeqabaqabeGadaaakeaacuWGLbqzgaqcamaaDaaaleaatCvAUfeBSjuyZL2yd9gzLbvyNv2CaeHbwvMCKfMBHbaceiGaa8xmaaqaaiabdkgaIbaakiabg2da9iqbdwgaLzaajaWaa0baaSqaaiaa=fdaaeaacqWGIbGyaaGccaWFOaGaemOta40aaSbaaSqaaiabdseaejabcYcaSaqabaGccqWGobGtdaWgaaWcbaGaemivaqLaa8xmaaqabaGccqGGSaalcqWGobGtdaWgaaWcbaGaemiDaqNaa8xmaaqabaGccqGGSaalcqWGebarcaWFPaGaaCzcaiaaxMaacqqGfbqrcqqGXbqCcqqGUaGlcqqGGaaicqqGOaakcqqGZaWmcqqGPaqkaaa@55A2@

where *b *= 1, 2,..., *N*_*b*_. The error rate estimates are used to compute data set specific estimates of the mean *m*_*dt1 *_and variance *σ*_*dt1*_^*2*^:

m^dt1(ND,NT1,Nt1,D)=1Nb∑b=1Nbe^1b     Eq. (4)
 MathType@MTEF@5@5@+=feaafiart1ev1aaatCvAUfKttLearuWrP9MDH5MBPbIqV92AaeXatLxBI9gBaebbnrfifHhDYfgasaacH8akY=wiFfYdH8Gipec8Eeeu0xXdbba9frFj0=OqFfea0dXdd9vqai=hGuQ8kuc9pgc9s8qqaq=dirpe0xb9q8qiLsFr0=vr0=vr0dc8meaabaqaciaacaGaaeqabaqabeGadaaakeaacuWGTbqBgaqcamaaBaaaleaacqWGKbazcqWG0baDcqaIXaqmaeqaaOWaaeWaaeaacqWGobGtdaWgaaWcbaGaemiraqeabeaakiabcYcaSiabd6eaonaaBaaaleaacqWGubavcqaIXaqmaeqaaOGaeiilaWIaemOta40aaSbaaSqaaiabdsha0jabigdaXaqabaGccqGGSaalcqWGebaraiaawIcacaGLPaaacqGH9aqpdaWcaaqaaiabigdaXaqaaiabd6eaonaaBaaaleaacqWGIbGyaeqaaaaakmaaqahabaGafmyzauMbaKaadaqhaaWcbaGaeGymaedabaGaemOyaigaaaqaaiabdkgaIjabg2da9iabigdaXaqaaiabd6eaonaaBaaameaacqWGIbGyaeqaaaqdcqGHris5aOGaaCzcaiaaxMaacqqGfbqrcqqGXbqCcqqGUaGlcqqGGaaicqqGOaakcqqG0aancqqGPaqkaaa@598A@

and

σ^dt12(ND,NT1,Nt1,D)=1Nb−1∑b=1Nb(e^1b−m^dt1)2.     Eq. (5)
 MathType@MTEF@5@5@+=feaafiart1ev1aaatCvAUfKttLearuWrP9MDH5MBPbIqV92AaeXatLxBI9gBaebbnrfifHhDYfgasaacH8akY=wiFfYdH8Gipec8Eeeu0xXdbba9frFj0=OqFfea0dXdd9vqai=hGuQ8kuc9pgc9s8qqaq=dirpe0xb9q8qiLsFr0=vr0=vr0dc8meaabaqaciaacaGaaeqabaqabeGadaaakeaaiiGacuWFdpWCgaqcamaaDaaaleaacqWGKbazcqWG0baDcqaIXaqmaeaacqaIYaGmaaGcdaqadaqaaiabd6eaonaaBaaaleaacqWGebaraeqaaOGaeiilaWIaemOta40aaSbaaSqaaiabdsfaujabigdaXaqabaGccqGGSaalcqWGobGtdaWgaaWcbaGaemiDaqNaeGymaedabeaakiabcYcaSiabdseaebGaayjkaiaawMcaaiabg2da9maalaaabaGaeGymaedabaGaemOta40aaSbaaSqaaiabdkgaIbqabaGccqGHsislcqaIXaqmaaWaaabCaeaadaqadaqaaiqbdwgaLzaajaWaa0baaSqaaiabigdaXaqaaiabdkgaIbaakiabgkHiTiqbd2gaTzaajaWaaSbaaSqaaiabdsgaKjabdsha0jabigdaXaqabaaakiaawIcacaGLPaaadaahaaWcbeqaaiabikdaYaaaaeaacqWGIbGycqGH9aqpcqaIXaqmaeaacqWGobGtdaWgaaadbaGaemOyaigabeaaa0GaeyyeIuoakiabc6caUiaaxMaacaWLjaGaeeyrauKaeeyCaeNaeeOla4IaeeiiaaIaeeikaGIaeeynauJaeeykaKcaaa@66A1@

For infinitely large sizes of *N*_*D *_and *N*_*T1 *_the observed variance *σ*_*dt1*_^*2 *^equals the true variance. Therefore, without any loss of generality, Eq. (5) can be written as

σ^dt12=σd12+w(1ND,1NT1,1Nt1,D)     Eq. (6)
 MathType@MTEF@5@5@+=feaafiart1ev1aaatCvAUfKttLearuWrP9MDH5MBPbIqV92AaeXatLxBI9gBaebbnrfifHhDYfgasaacH8akY=wiFfYdH8Gipec8Eeeu0xXdbba9frFj0=OqFfea0dXdd9vqai=hGuQ8kuc9pgc9s8qqaq=dirpe0xb9q8qiLsFr0=vr0=vr0dc8meaabaqaciaacaGaaeqabaqabeGadaaakeaaiiGacuWFdpWCgaqcamaaDaaaleaacqWGKbazcqWG0baDcqaIXaqmaeaacqaIYaGmaaGccqGH9aqpcqWFdpWCdaqhaaWcbaGaemizaqMaeGymaedabaGaeGOmaidaaOGaey4kaSIaem4DaC3aaeWaaeaadaWcaaqaaiabigdaXaqaaiabd6eaonaaBaaaleaacqWGebaraeqaaaaakiabcYcaSmaalaaabaGaeGymaedabaGaemOta40aaSbaaSqaaiabdsfaujabigdaXaqabaaaaOGaeiilaWYaaSaaaeaacqaIXaqmaeaacqWGobGtdaWgaaWcbaGaemiDaqNaeGymaedabeaaaaGccqGGSaalcqWGebaraiaawIcacaGLPaaacaWLjaGaaCzcaiabbweafjabbghaXjabb6caUiabbccaGiabbIcaOiabbAda2iabbMcaPaaa@55D1@

where *w *is a data set dependent small sample effect term that vanish for large data set sizes. To estimate the first term, a first order approximation is introduced, yielding

σ^dt12(1ND,1NT1,1Nt1,D)=α0(D)+α1(D)ND+α2(D)NT1+α3(D)Nt1     Eq. (7)
 MathType@MTEF@5@5@+=feaafiart1ev1aaatCvAUfKttLearuWrP9MDH5MBPbIqV92AaeXatLxBI9gBaebbnrfifHhDYfgasaacH8akY=wiFfYdH8Gipec8Eeeu0xXdbba9frFj0=OqFfea0dXdd9vqai=hGuQ8kuc9pgc9s8qqaq=dirpe0xb9q8qiLsFr0=vr0=vr0dc8meaabaqaciaacaGaaeqabaqabeGadaaakeaaiiGacuWFdpWCgaqcamaaDaaaleaacqWGKbazcqWG0baDcqaIXaqmaeaacqaIYaGmaaGcdaqadaqaamaalaaabaGaeGymaedabaGaemOta40aaSbaaSqaaiabdseaebqabaaaaOGaeiilaWYaaSaaaeaacqaIXaqmaeaacqWGobGtdaWgaaWcbaGaemivaqLaeGymaedabeaaaaGccqGGSaaldaWcaaqaaiabigdaXaqaaiabd6eaonaaBaaaleaacqWG0baDcqaIXaqmaeqaaaaakiabcYcaSiabdseaebGaayjkaiaawMcaaiabg2da9iab=f7aHnaaBaaaleaacqaIWaamaeqaaOGaeiikaGIaemiraqKaeiykaKIaey4kaSYaaSaaaeaacqWFXoqydaWgaaWcbaGaeGymaedabeaakiabcIcaOiabdseaejabcMcaPaqaaiabd6eaonaaBaaaleaacqWGebaraeqaaaaakiabgUcaRmaalaaabaGae8xSde2aaSbaaSqaaiabikdaYaqabaGccqGGOaakcqWGebarcqGGPaqkaeaacqWGobGtdaWgaaWcbaGaemivaqLaeGymaedabeaaaaGccqGHRaWkdaWcaaqaaiab=f7aHnaaBaaaleaacqaIZaWmaeqaaOGaeiikaGIaemiraqKaeiykaKcabaGaemOta40aaSbaaSqaaiabdsha0jabigdaXaqabaaaaOGaaCzcaiaaxMaacqqGfbqrcqqGXbqCcqqGUaGlcqqGGaaicqqGOaakcqqG3aWncqqGPaqkaaa@70DC@

where *α*_*0*_*(D) *≈ *σ*_*d1*_^*2*^*(N*_*d*_*)*.

By performing multiple repeated random sampling sessions (each consisting of *N*_*b *_repeated designs and tests as described earlier) for different combinations of data set sizes and the sizes of the design and tests sets, it is possible to estimate the coefficients *α*_*i*_*(D), i *= 0,1, 2, 3, in Eq. (7) by multivariate least squares fitting. With access to the fitted coefficient *α*_*0*_*(D)*, an estimate of the desired quantity *σ*_*d1 *_^*2*^*(N*_*d*_*) *is obtained.

Preliminary simulation results indicated that the coefficient *α*_*1 *_in Eq. (7) was relatively small. In other words, the size *N*_*D *_of the bag of design examples did not seem to have any large contribution to the bias. Therefore the results presented in this work were based on the simplified model:

σ^dt12(1NT,1Nt,D)≈α0(D)+α1(D)NT1+α2(D)Nt1.     Eq. (8)
 MathType@MTEF@5@5@+=feaafiart1ev1aaatCvAUfKttLearuWrP9MDH5MBPbIqV92AaeXatLxBI9gBaebbnrfifHhDYfgasaacH8akY=wiFfYdH8Gipec8Eeeu0xXdbba9frFj0=OqFfea0dXdd9vqai=hGuQ8kuc9pgc9s8qqaq=dirpe0xb9q8qiLsFr0=vr0=vr0dc8meaabaqaciaacaGaaeqabaqabeGadaaakeaaiiGacuWFdpWCgaqcamaaDaaaleaacqWGKbazcqWG0baDcqaIXaqmaeaacqaIYaGmaaGcdaqadaqaamaalaaabaGaeGymaedabaGaemOta40aaSbaaSqaaiabdsfaubqabaaaaOGaeiilaWYaaSaaaeaacqaIXaqmaeaacqWGobGtdaWgaaWcbaGaemiDaqhabeaaaaGccqGGSaalcqWGebaraiaawIcacaGLPaaacqGHijYUcqWFXoqydaWgaaWcbaGaeGimaadabeaakiabcIcaOiabdseaejabcMcaPiabgUcaRmaalaaabaGae8xSde2aaSbaaSqaaiabigdaXaqabaGccqGGOaakcqWGebarcqGGPaqkaeaacqWGobGtdaWgaaWcbaGaemivaqLaeGymaedabeaaaaGccqGHRaWkdaWcaaqaaiab=f7aHnaaBaaaleaacqaIYaGmaeqaaOGaeiikaGIaemiraqKaeiykaKcabaGaemOta40aaSbaaSqaaiabdsha0jabigdaXaqabaaaaOGaeiOla4IaaCzcaiaaxMaacqqGfbqrcqqGXbqCcqqGUaGlcqqGGaaicqqGOaakcqqG4aaocqqGPaqkaaa@635C@

### The 2-dimensional normal distributions

Two probes from the colon cancer data set [9] with accession numbers R87126 and X57351 corresponding to the genes *Nonmuscle Type A Myosin Heavy Chain (NMMHC-A) *and *Interferon induced transmembrane protein 2 (IFITM2) *which can be used for a reasonable discrimination between the two classes considered were selected for the definition of two-dimensional sample distributions for two different classes. The following estimates of the mean vectors m_i _and covariance matrices Σ_i _were obtained for the two genes used: m_1 _= (0.7889, -0.36883), m_2 _= (-0.4339, 0.2028), Σ_1 _= (1.5598, 0.4208; 0.4208, 0.6045) and Σ_2 _= (0.1800, 0.1027; 0.1027, 1.1197). In the simulations, a pair of two-dimensional normal distribution with these parameters was used to generate the examples needed.

### Simulation procedure for estimation of m_*d1 *_and *σ*_*d1*_^*2 *^in Eq. (1)

First, 50 independent design bags of size *N*_*D *_= 100 with equal number of samples from class 1 and class 2 and 50 corresponding test bags with *N*_*T1 *_= 25 samples from class 1, were generated from the 2-dimensional normal distributions. Then for each pair of bags, *N*_*b *_= 1,000 different designs, each with *N*_*d *_samples drawn with replacement, were implemented. These classifiers do not include any feature selection and used the same Fisher's linear discriminant classification algorithm that was used for the colon data. Each classifier was tested using different sizes *N*_*t1 *_of the test sets. Multivariate least square fitting was used to obtain 1,000 different values for *α*_0_*(D) *(see Additional File 2). The value of *N*_*T1 *_was then increased, *N*_*T1 *_= 50, 75,..., 200, yielding histograms for seven additional sizes of the test bag. The mean value and a two-sided 95% CI for the eight histograms were calculated. The true *m*_*d1 *_and *σ*_*d1 *_^*2 *^were obtained by testing 10,000 independently designed classifiers using 500,000 test samples.

### Variance estimation for independent data

Monte-Carlo simulations were performed to verify the linear mapping between *1/N*_*t *_and *σ*_*dt *_^*2 *^in Eq. (2). We determined *σ*_*dt *_^*2 *^for different values of *N*_*t *_and *N*_*d *_for a Fisher linear discriminant classifier where equal number of samples from class1 and class 2 were drawn for design and testing. The samples were drawn from two 8-dimensional normal distributions with means m_1 _= [0,0,0,0,0,0,0,0]^T ^and m_2 _= [2.56, 0, 0, 0, 0, 0, 0, 0]^T ^where T denotes the transpose operator. The covariance matrix used was the identity matrix. Please note that the probabilities of encountering a sample of class 1 or class 2 are not used here. This statistical model is a nontrivial model suitable for simulation based validation of our theoretical results in Figure 3. The values of *N*_*d *_considered were *N*_*d *_= 20, 30, 40 and the number of test samples used for each value of *N*_*d *_were *N*_*t *_= 20, 30,..., 90, 100. Each point (value of *σ*_*dt *_^*2*^) was obtained using a histogram of 1,000 independent point estimates. To verify the results, 1,000 separate and independent high accuracy point estimates of *m*_*d *_and *σ*_*d*_^*2 *^were computed, each using 10,000 test samples for varying design set sizes *N*_*d*_.

## List of abbreviations

• *α *_*i *_– coefficients for the first order approximation of the variance model, *i *= 0,1, 2, 3

• CI – Confidence Interval

• *D *– a dataset with *N *samples

• *m*_*d *_– mean error (misclassification) rate based on design with *N*_*d *_design samples and test with a large number (infinity) of test samples

• *m*_*dt *_– mean error rate based on *N*_*d *_design samples and *N*_*t *_test samples

• *N *– total number of samples used

• *N*_*b *_– number of times a procedure is carried out

• *N*_*d *_– number of samples used for design

• *N*_*D *_– number of samples used in design bag

• *N*_*t *_– number of samples used for test

• *N*_*T *_– number of samples used in test bag

• pdf – probability density function

• RIDT – Repeated Independent Design and Test

• RRS – Repeated Random Sampling

• *σ*_*d*_^*2 *^– variance of the error rate distribution from design with *N*_*d *_samples

• *σ*_*dt *_^*2 *^– variance of the error rate distribution from design with *N*_*d *_samples and test with *N*_*t *_samples

## Authors' contributions

UWB implemented and evaluated the variance models and drafted the manuscript. HG and MF made intellectual contributions to the work and were involved in manuscript preparations. MG derived the variance models and participated in the implementation and evaluation. AI participated in the implementation and evaluation. AI and MG supervised the study. All authors read and approved the final manuscript.

## Supplementary Material

Additional File 1 The pdf-file contains the derivation of Eq. (2). Click here for file

Additional File 2 The pdf-file contains a more detailed description of the implementation of the RIDT procedure. Click here for file
