# Supplementary material for: Improved variance estimation of classification performance via reduction of bias caused by small sample size
Source: BMC Bioinformatics. 2006 Mar 13;7:127. doi: 10.1186/1471-2105-7-127 (PMC1435937; doi:10.1186/1471-2105-7-127)
Supplement: Additional File 1 — The pdf-file contains the derivation of Eq. (2). [file 1471-2105-7-127-S1.pdf]

Additional File 1

Derivation of Eq. (2)

The derivation presented in this paper employs the identity

$$p(k_t|N_t, N_d) = \int_0^1 P(k_t|e, N_t)p(e|N_d)de. \quad (1)$$

with $P(k_t|e, N_t)$ defined as the binomial distribution with mean N_te and variance $N_te(1-e)$. $p(e|N_d)$ is the conditional pdf (prior) of the error rate given that the classifier has been designed using N_d examples. Using this identity one immediately finds that

$$< k_t|N_t, N_d >= \sum_{k_t} k_tP(k_t|N_t, N_d) = \quad (2)$$

$$\int_0^1 \sum_{k_t} k_tP(k_t|e, N_t)P(e|N_d)de =$$

$$\int_0^1 N_tep(e|N_d)de = N_tm_d. \quad (3)$$

where

$$m_d = \int_0^1 ep(e|N_d)de =< e|N_d > \quad (4)$$

is the conditional mean value of the error rate given that the classifier has been designed using N_d examples. Similarly

$$< k_t^2|N_t, N_d >= \sum_{k_t} k_t^2P(k_t|N_t, N_d) =$$

$$\int_0^1 \sum_{k_t} k_t^2P(k_t|e, N_t)P(e|N_d)de =$$

$$\int_0^1 (N_te(1-e) + (N_te)^2)p(e|N_d)de =$$

$$\int_0^1 N_t(e + (N_t-1)e^2)p(e|N_d)de =$$

$$N_tm_d + N_t(N_t-1)(\sigma_d^2 + m_d^2). \quad (5)$$

Thus

$$< (k_t - N_tm_d)^2|N_t, N_d >=$$

$$N_tm_d + N_t(N_t-1)(\sigma_d^2 + m_d^2) - (N_tm_d)^2 =$$

$$N_t(m_d + N_t\sigma_d^2 - \sigma_d^2 - m_d^2). \quad (6)$$

From above it is straight forward to find the expression for the mean as

$$m_{td} =< \hat{e}|N_d, N_t >=< k_t/N_t|N_d, N_t >= m_d. \quad (7)$$

This result shows, as already discussed by Fukunaga and Hayes [1] that the mean value only depends on the design step, the testing does not introduce any additional bias. Moreover one also finds

$$\sigma_{td}^2 =< (\hat{e} - m_{td})^2|N_d, N_t >=$$

$$< (k_t/N_t - m_d)^2|N_d, N_t >=$$

$$\frac{1}{N_t^2} < (k_t - N_tm_d)^2|N_d, N_t >=$$

$$\frac{1}{N_t^2} N_t(m_d + N_t\sigma_d^2 - \sigma_d^2 - m_d^2) \tag{8}$$

which yields the expression

$$\sigma_{td}^2 = \sigma_d^2(1 - 1/N_t) - \frac{m_d(m_d - 1)}{N_t}. \tag{9}$$

that can be rewritten to the desired expression

$$\sigma_{td}^2 = \sigma_d^2 + \frac{m_d(1 - m_d) - \sigma_d^2}{N_t}. \tag{10}$$

This expression clearly shows how the observed variance depends on the number of test examples and on the mean and variance associated with the design step. For example one immediately confirms the intuitive behavior that, when N_t becomes large, the variance contribution comes only from the design step.

One may also note that the above expression can be written

$$\sigma_{td}^2 = \sigma_d^2 + \frac{m_d(1 - m_d) - \sigma_d^2}{N_t}. \tag{11}$$

and that

$$m_d(1 - m_d) - \sigma_d^2 = m_d - (\sigma_d^2 + m_d^2) =$$

$$< \hat{e}|N_d > - < \hat{e}^2|N_d >= \int_0^1 \hat{e}(1 - \hat{e})de \geq 0 \tag{12}$$

since $\hat{e} \in [0, 1]$. This shows the intuitive but important fact that

$$\sigma_{td} \geq \sigma_d \tag{13}$$

which means that the resulting confidence intervals will be conservative if σ_{td} is used.

References

[1] K. Fukunaga and R.R. Hayes, **Estimation of Classifier Performance.** *IEEE Transactions on Pattern Analysis and Machine Learning* 1989, **11:**1087-1101.
