# Supplementary material for: Improved variance estimation of classification performance via reduction of bias caused by small sample size
Source: BMC Bioinformatics. 2006 Mar 13;7:127. doi: 10.1186/1471-2105-7-127 (PMC1435937; doi:10.1186/1471-2105-7-127)
Supplement: Additional File 2 — The pdf-file contains a more detailed description of the implementation of the RIDT procedure. [file 1471-2105-7-127-S2.pdf]

Additional File 2

Implementation of the RIDT used for the simulations

In order to determine the coefficients α_i in Eq. (8) of the paper, the values of N_T and N_t have to be varied independently. As explained here, this is achieved by first cutting out fractions of the original bag of test examples into smaller test bags of sizes $N_T^{(r_i)}$, $i = 1, 2, 3, 4$ and then cutting out fractions of each test bag as individual test sets of sizes $N_t^{(r_{ij})}$, $j = 1, 2, 3, 4, 5$. Here we use the same number of samples in both classes and the number of samples in the design, N_d, is the same as in the design bag, N_D.

1. Create a design bag containing N_D samples and a test bag containing N_T samples. Together the bags constitute the total data set, D.
2. Use constrained regression to obtain estimates of σ_{d1}^2 (similarly for class 2) as follows:

- Cut out four different test bags containing $N_T^{(r_1)} = N_T$, $N_T^{(r_2)} = 0.75N_T$, $N_T^{(r_3)} = 0.5N_T$, and $N_T^{(r_4)} = 0.25N_T$ examples.
- For each test bag of size $N_T^{(r_i)}$, $i = 1, 2, 3, 4$, perform the following steps $N_b = 1000$ times:
  - (a) Resample $N_D/2$ examples for each class from the original design bag and use them to design a classifier.
  - (b) Cut out test sets consisting of $N_t^{(r_{i1})} = N_T^{(r_i)}$, $N_t^{(r_{i2})} = 0.9N_T^{(r_i)}$, $N_t^{(r_{i3})} = 0.8N_T^{(r_i)}$, $N_t^{(r_{i4})} = 0.7N_T^{(r_i)}$, and $N_t^{(r_{i5})} = 0.6N_T^{(r_i)}$ examples without replacement. Thus, for each size $N_T^{(r_i)}$, $i = 1, 2, 3, 4$ of the test bag, five different sizes $N_t^{(r_{ij})}$, $j = 1, 2, 3, 4, 5$ of the test set are created.
  - (c) Test the classifier with the different test sets and store the error rate estimates $\hat{e}_1(N_D, N_T^{(r_i)}, N_t^{(r_{ij})})$, $i = 1, 2, 3, 4$ and $j = 1, 2, 3, 4, 5$.
  - (d) Permute (scramble) the sample order inside the test bag of size $N_T^{(r_i)}$.
- Using the $N_b = 1000$ estimates $\hat{e}_1(N_D, N_d, N_T^{(r_i)}, N_t^{(r_{ij})})$ obtained for $i = 1, 2, 3, 4$ and $j = 1, 2, 3, 4, 5$, compute the 20 mean estimates $\hat{m}_{d1}(N_D, N_T^{(r_i)}, N_t^{(r_{ij})})$ and 20 variance estimates $\hat{\sigma}_{d1}^2(N_D, N_T^{(r_i)}, N_t^{(r_{ij})})$.
- Using the 20 variance estimates perform constrained multivariate linear regression to obtain and estimate of σ_{d1}^2.
- Using the 20 mean estimates, compute the arithmetic mean as an estimate of m_{d1}.
